# Impact of reactive oxygen species (ROS) on the control of parasite loads and inflammation in *Leishmania amazonensis* infection

**DOI:** 10.1186/s13071-016-1472-y

**Published:** 2016-04-07

**Authors:** Eric Henrique Roma, Juan Pereira Macedo, Grazielle Ribeiro Goes, Juliana Lauar Gonçalves, Waldionê de Castro, Daniel Cisalpino, Leda Quercia Vieira

**Affiliations:** Departamento de Bioquímica e Imunologia, Instituto de Ciências Biológicas, Universidade Federal de Minas Gerais, Belo Horizonte, MG Brazil; Departamento de Microbiologia, Instituto de Ciências Biológicas, Universidade Federal de Minas Gerais, Belo Horizonte, MG Brazil; Current address: Instituto Nacional de Infectologia, Fiocruz, Rio de Janeiro, RJ Brazil; Current address: Laboratory of Malaria and Vector Research, Vector Molecular Biology Section, National Institutes of Health, NIAID, Rockville, MD USA

**Keywords:** *Leishmania amazonensis*, ROS, NOX2, Neutrophils, Inflammation

## Abstract

**Background:**

Reactive oxygen species (ROS) protect the host against a large number of pathogenic microorganisms. ROS have different effects on parasites of the genus *Leishmania*: some parasites are susceptible to their action, while others seem to be resistant. The role of ROS in *L. amazonensis* infection *in vivo* has not been addressed to date.

**Methods:**

In this study, C57BL/6 wild-type mice (WT) and mice genetically deficient in ROS production by phagocytes (gp91^phox−/−^) were infected with metacyclic promastigotes of *L. amazonensis* to address the effect of ROS in parasite control. Inflammatory cytokines, parasite loads and myeloperoxidase (MPO) activity were evaluated. In parallel, *in vitro* infection of peritoneal macrophages was assessed to determine parasite killing, cytokine, NO and ROS production.

**Results:**

*In vitro* results show induction of ROS production by infected peritoneal macrophages, but no effect in parasite killing. Also, ROS do not seem to be important to parasite killing *in vivo*, but they control lesion sizes at early stages of infection. IFN-γ, TNF-α and IL-10 production did not differ among mouse strains. Myeloperoxidase assay showed augmented neutrophils influx 6 h and 72 h post - infection in gp91^phox−/−^ mice, indicating a larger inflammatory response in gp91^phox−/−^ even at early time points. At later time points, neutrophil numbers in lesions correlated with lesion size: larger lesions in gp91^phox−/−^ at earlier times of infection corresponded to larger neutrophil infiltrates, while larger lesions in WT mice at the later points of infection also displayed larger numbers of neutrophils.

**Conclusion:**

ROS do not seem to be important in *L. amazonensis* killing, but they regulate the inflammatory response probably by controlling neutrophils numbers in lesions.

## Background

Leishmaniasis are a spectrum of diseases caused by parasites of the genus  *Leishmania*. This disease is endemic in 88 countries and affects two million people every year. The diseases may present themselves as cutaneous, mucocutaneous or visceral forms, depending on which species is involved in the infection [1].

In the well-established model of infection of mice with *L. major,* resistance to several parasite strains is mediated by the development of a Th1 immune response, while susceptibility is characterized by the development of a Th2 response [2–4], or to production of IL-10 [5]. However, C57BL/6 and C57BL/10 mice, which completely heal infection with *L. major*, develop chronic non-healing lesions when infected with *L. amazonensis* [6–8]. In addition, this susceptibility is independent of a Th2 response [6].

Macrophages are the main host cell for *Leishmania* spp. When infected with these parasites, macrophages sustain *Leishmania* spp. growth. However, when activated to produce nitric oxide, macrophages can kill *L. major* [9, 10]. Activation of macrophages is dependent on IFN-γ and TNF [9, 10]. Macrophages infected with *L. amazonensis* produce less TNF, even in the presence of IFN-γ [11, 12]. Hence, activation of *L. amazonensis* infected macrophages is deficient, at least *in vitro* [11, 12].

Reactive oxygen species derive from oxygen reduction, generating a group of highly reactive ions, molecules and radicals. ROS may be generated in mitochondria as respiratory chain products [13] and also participate in many biological processes, such as hormonal biosynthesis [14], cellular signalling [15] and destruction of intracellular pathogens [16]. ROS are also important effector agents against intracellular pathogens, induced by IFN-γ or Toll-like receptors [17, 18].

Phagocyte NADPH oxidases (NOX2) are a group of multimeric proteins composed by cytosolic chains (p67 ^phox^, p47 ^phox^ and p40 ^phox^), a small G protein (rac1 or rac2) and membrane-associated subunits (gp91 ^phox^ and p22 ^phox^) [19]. As other isoforms of NADPH oxidases, NOX2 catalyzes the production of superoxide anion (O_2_^●−^) by reducing oxygen, using NADPH as the electron donor [20]. The resulting superoxide may generate many reactive species including oxidized halogens, oxygen singlet and other free radicals. Phagocytic cells use these oxidants to kill intracellular pathogens, but these species can also cause tissue damage to host cells. Hence, NOX2 is strictly regulated and is activated upon specific stimuli, such as phagocytosis triggered by pathogen-associated molecular patterns (PAMPS) [21].

Gp91^phox^ is essential for NOX2 function. It is responsible for molecular oxygen reduction by electrons provided by NADPH [22]. Humans or mice deficient in gp91^phox^ present X-linked chronic granulomatous disease (CGD) [23, 24]. Patients with CGD have recurrent infections that can cause death as early as childhood. Although chemotaxis, degranulation and phagocytosis are normal, CGD patients show deficiency in destruction of phagocytosed microorganisms due to lack of metabolites generated from superoxide [25]. Accordingly, gp91^phox^ knockout mice eventually develop CGD. These mice respond to chemically induced peritonitis with extensive neutrophil infiltration [23], and increased secretion of inflammatory cytokines and chemokines during lung infection by pneumococcal pneumonia [26].

The effect of ROS in *in vivo* infection caused by *Leishmania* spp. has been less well studied, since nitric oxide is believed to be the major effector molecule involved in parasite killing [27, 28]. *In vitro* studies show an irrelevant role of ROS in parasite killing by macrophages infected with *L. major* [29, 30] and *L. guyanensis* [31]. *In vivo* ROS control *L. major* parasitism in mice [30]. In *L. donovani* infection, ROS would be important only for short-term control of the parasites [32]. These differences in resistance to ROS observed during infection with different species of *Leishmania* make it necessary to investigate the role of ROS in other *Leishmania* spp. A few papers have addressed the role of ROS during infection with *L. amazonensis.* The role of ROS has been addressed *in vitro* by some authors by measuring the amount of ROS produced by macrophages infected with *L. amazonensis* [11, 33] or with *L. pifanoi*, a parasite belonging to the Mexicana complex, like *L. amazonensis* [34]. ROS play a role in parasite killing of *L. amazonensis* by activated macrophages (with both IFN-γ and LPS) treated with ERK inhibitor [35]. However, the role of ROS produced upon phagocytosis of *L. amazonensis* on parasite killing and during *invivo* infection has not yet been addressed.

## Methods

### Mice and ethics statements

C57BL/6 mice were obtained from the animal house of the Instituto de Ciências Biológicas, Universidade Federal de Minas Gerais (CEBIO). Mice which genes for gp91^phox^ subunit of NADPH oxidase were deleted by homologue recombination (B6.129S-Cybb^tm1Din^/J, here named gp91^phox−/−^) [23] were purchased from Jackson Farms (Glensville, NJ, USA). Animals were kept in conventional conditions with barriers, controlled light cycle and temperature. Food and water were provided *ad libitum*. All animals used in this study were 6 to 12 week- old. This project was approved by the local ethical committee under the protocol CETEA 031/09.

### Parasites, infections and generation of Leishmania antigen

*Leishmania amazonensis* (IFLA/BR/67/PH8) was maintained in Grace’s medium as previously described [36]. Metacyclic promastigotes were purified in a ficoll gradient [37], washed, resuspended in phosphate buffered saline (PBS, pH 7.3) and counted. Inocula of 1 × 10^6^ parasites/40 μl of PBS were injected in the mouse left hind footpad. Lesion development was followed by measuring the thickness of the footpad swelling using a digital micrometer (Starrett 727, Itu, SP, Brazil). Antigens were prepared from log phase promastigotes, washed in PBS and submitted to seven cycles of freezing in liquid nitrogen and thawing (37 °C). Suspensions were adjusted to a final concentration of 1 mg of protein/ml and kept at −70 °C until use. Protein concentration was assessed by the Lowry assay [38].

### Quantification of parasites

Mice were sacrificed by cervical dislocation. The footpads were removed and disinfected in 70 % ethanol for 5 min and air - dried in the laminar flow hood. The footpads were cut in small parts and placed in RPMI medium (GIBCO, Grand Island, NY, USA) containing 100U/ml penicillin, 100 μg/ml streptomycin (GIBCO) and 125U/ml collagenase A (Sigma-Aldrich, Inc, St. Louis, MO, USA) for 2 h at 37 °C in a humidified chamber and atmosphere containing 5 % CO_2_. After incubation the pieces of footpads were ground, filtered with a 40 μm cell strainer filter (BD Falcon, Franklin Lakes, NJ, USA) and washed with 10 ml of RPMI 0.05 % DNAse (Sigma-Aldrich). The homogenates were centrifuged at 50 × *g* for 4 min to remove large tissue debris and the supernatants were collected and centrifuged at 1,500 × *g* for 15 min. The sediment was re-suspended in 1 ml of complete RPMI (GIBCO) (RPMI supplemented with 10 % heat-inactivated fetal bovine serum (FBS) (Cultilab, Campinas, SP, Brazil), 100U/ml penicillin, 100 μg/ml streptomycin and 2 mM l-glutamine (GIBCO BRL). Fifty microliters of the suspension were serially diluted in a 96-well plate containing 150 μl of Grace’s insect medium supplemented with 20 % heat-inactivated FBS, 100U/ml penicillin, 100 μg/ml streptomycin and 2 mM l-glutamine (GIBCO) (Grace’s complete medium) in each well. The samples were serially diluted in Grace’s complete medium (1:4) in triplicates. Pipette tips were discarded after each dilution. Plates were cultured for 10 days in BOD incubator at 25 °C and the last positive dilution was registered as the titre. Results are expressed as the negative logarithm of the titre.

### Luminometry assay

Mice were injected intraperitoneally with 2 ml of 4 % thioglycollate (BD Biosciences, Franklin Lakes, NJ, USA). After 3 days, mice were euthanized and the peritoneum cells were harvested by repeated cycles of aspiration and re-injection with 10 ml of cold PBS in a 10 ml syringe with a 24G needle. Considering cell morphology and adherence, more than 80 % of the cells harvested were macrophages. Cells were centrifuged at 4 °C, 1,500 × *g* for 10 min, counted in a hemocytometer and the concentration was adjusted to 1×10^6^ cells/100 μL of complete RPMI without phenol red. Cells (1 × 10^6^ cells/well) were plated in 96 well opaque plates (NUNC, Rochester, NY, USA) together with 0.05 mM of luminol (5-amino-2,3-dihydro-1,4-phthalazinedione, Sigma-Aldrich). Immediately before the measurement, *L. amazonensis* metacyclic promastigotes were added in the proportion of 10 parasites per macrophage. The measurement was followed for 90 min with one minute of interval between the measurements. The production of ROS was assessed by the light intensity generated by the reaction between ROS and luminol and expressed as relative light units.

### *In vitro* assays for parasite burden

Macrophages were isolated from the peritoneal cavity of mice 3 days after injection of 2 ml of 4 % thioglycollate medium (BD Biosciences, Franklin Lakes, NJ, USA) into the peritoneal cavity. After this time, mice were euthanized and the peritoneum cells were harvested by repeated cycles of aspiration and re-injection with 10 ml of cold PBS in a 10 ml syringe with a 24G needle. More than 80 % of the cells harvested were macrophages. The cells were centrifuged at 4 °C, 1,500 × *g* for 10 min and re-suspended in DMEM supplemented with 10 % fetal bovine serum (FBS) (Cultilab, Campinas, SP, Brazil), 1 % penicillin-streptomycin and 2 mM l-glutamine. Macrophages were counted in a hemocytometer prior to seeding 5×10^5^ cells into each well of a 24-well plate and incubated at 37 °C, 5 % CO_2_ for 2 h. After this time, *L. amazonensis* metacyclic promastigotes were added in the proportion of 5 parasites per macrophage during 4 h. After this period, cells were washed three times with phosphate-buffered saline (PBS, pH 7.3) to remove extracellular parasites. Cells were fixed or reincubated with medium for 72 h before fixation with methanol. Coverslips with attached macrophages were stained with Panótico (Laborclin, Pinhais, PR, Brazil) and a minimum of 200 macrophages per coverslip were counted. The results were expressed as an infection index (% infected macrophages x number of amastigotes/total number of macrophages). The following drugs were used in these assays: apocynin (APO) (300 μM; Sigma-Aldrich) and N-acetyl-cysteine (NAC) (1 mM; Sigma-Aldrich) and H_2_O_2_ (100 μM). Drugs were added to the cells 2 h before and immediately after infection.

### Real time PCR

Total RNA, obtained from lesions at 4, 8, 12 and 16 weeks post-infection, was extracted using Trizol reagent (Invitrogen, Carlsbad, CA, USA) according to the manufacturer’s instructions. One μg of total RNA obtained from the lesions or lymph nodes was reverse transcribed using reverse transcriptase (Promega, Southampton, UK) and oligo (dT) 15-mer primer (Promega, Southampton, UK). PCR amplification was performed with a programmable thermal cycler (Perkin–Elmer 2400, Waltham, MA, USA). The cDNA amplification protocol was as follows: 2 min at 50 °C, activation of AmpliTaq at 95 °C for 10 min, melting at 95 °C for 15 s. For the annealing and final extension, the samples were heated at 60 °C for 1 min for 45 cycles. For dissociation curve, the samples were heated at 95 °C for 15 s, following cooling at 60 °C for 5 s. Finally, the samples were cooled for 1 min at 4 °C.

The amplification of cDNA was made using specific primers as follow: IFN-γ (forward TCAAGTGGCATAGATGTGGAAGAA, reverse TGGCTCTGCAGGATTTTCATG), IL-4 (forward ACAGGAGAAGGGACGCCA, reverse GAAGCCCTACAGACGAGCTCA), IL-10 (forward GGTTGCCAATTATCGGA, reverse ACCTGCTCCACTGCCTTGCT), TNF-α (forward TTCTGTCTACTGAACTTCGGGGTGATCGGTCC, reverse GTATGAGATAGCAAATCGGCTGACGGTGTGGG), IL1-β (forward CAACCAACAAGTGATATTCTCCAT, reverse GATCCACACTCTCCAGCTGCA), iNOS (forward CCCTTCCGAAGTTTCTGGCAGCAGC, reverse GGCTGTCAGAGCCTCGTGGCTTTGG). The reactions were developed in the ABI PRISM®7900HT (Applied Biosystems, Foster City, CA, USA) using 20 % of reaction in cDNA volume and 15 μl of total PCR mixture. All reactions were performed in duplicate using SYBR Green Master Mix (Applied Biosystems) according to manufacturer’s instructions. Finally, the samples were cooled for 1 min at 4 °C. The specific cDNAs were normalized according to the expression of ribosomal 18 s gene (forward TACCACATCCAAGAAGGCAG, reverse TGCCCTCCAATGGATCCTC) based in ΔCT calculation. The results were expressed as fold increase of target gene expression compared to ribosomal 18 s gene expression.

### Cytokine and nitrite assays

Single cell suspensions of draining lymph nodes (dLNs) from mice infected for 8, 12 and 16 weeks post-infection plated in 24 well plates at 5 × 10^6^ cells/mL (Nunclon, Nunc) and stimulated with *L. amazonensis* antigen (50 μg/ml) for 72 h at 37 °C and 5 % CO_2_. Concanavalin A was used as positive control (10 μg/ml) of cytokine production. The levels of IFN-γ, IL-10, IL-6, IL-17a were measured in supernatant using appropriate kits (BD OptEIA^TM^, BD Biosciences, Franklin Lakes, NJ, USA) following manufacturer's instructions. Detection levels were 12.5 pg/ml for all cytokines measured.

Nitrites were measured in supernatants of peritoneal macrophage cultures infected with *L. amazonensis*. Briefly, peritoneal macrophages were harvested as described above. The cells (5 × 10^5^) were plated in 48 well plates in 500 μl of complete RPMI. After 16 h, the cultures were washed to remove the non-adherent cells, and cells were activated with 50U/ml of IFN-γ (BD biosciences, Franklin Lakes, NJ, USA) and 100 ng/ml of LPS (Sigma-Aldrich) for 4 h at 37 °C and 5 % CO_2_. Following the activation, the cells were infected with *L. amazonensis* metacyclic promastigotes at 10 parasites per macrophage. After 4 h of infection, the cells were washed, re-stimulated with IFN-γ and LPS at the same concentrations used before and incubated for 48 h at 37 °C and 5 % CO_2_. The supernatants of *in vitro* infected macrophages were collected 48 h after infection and the levels of IL-6, TNF-α, IL-17A and CXCL-1, MCP-1 and IL-10 were measured using appropriate kits (BD OptEIA^TM^, BD Biosciences, Franklin Lakes, NJ, USA) following manufacturer's instructions.

After 48 h, 50 μL of supernatants were collected and used to quantify nitrite using the Griess method [39] in 96 well plates. After 10 min of reaction the plates were read in a plate reader (EZ read 400, Biochrom, Cambridge, UK) at 540 nm.

### Myeloperoxidase assay

Neutrophil accumulation in the infected footpads was measured by assaying myeloperoxidase (MPO) activity. Briefly, the footpads were infected as described in item 2.2. Six and 72 h post-infection, mice were euthanized, the footpads were removed and snap-frozen in liquid nitrogen. On thawing, the tissue (100 mg of tissue per 1.9 ml of buffer) was homogenized in pH 4.7 buffer (0.1 M NaCl, 0.02 M Na_3_PO_4_, 0.015 M sodium-ethylenediaminetetraacetic acid), centrifuged at 260 × *g* for 10 min and the pellet subjected to hypotonic lyses (15 ml of 0.2 % NaCl solution followed by 30 s of equal volume of a solution containing 1.6 % NaCl and 5 % glucose). After further centrifugation, the pellet was resuspended in 0.05 M sodium phosphate buffer (pH 5.4) containing 0.5 % hexadecyltrimethylammonium bromide and re-homogenized. One-ml aliquots of the suspension were transferred into 1.5 ml conical microtubes followed by three freeze-thaw cycles using liquid nitrogen. The aliquots were then centrifuged for 15 min at 10,000 × *g*, the pellet was re-suspended to 1 ml. Myeloperoxidase activity in the re-suspended pellet was assayed by measuring the change in optical density (OD) at 450 nm using tetramethylbenzidine (1.6 mM) and H_2_O_2_ (0.5 mM). Results were expressed as OD units.

### Statistical analysis

Statistical significance between groups was determined by the unpaired, two-tailed Student’s t test using Prism software (GraphPad, La Jolla, CA, USA). *P* values < 0.05 were considered significant.

## Results

### *Leishmania amazonensis* metacyclic promastigotes are capable of inducing a respiratory burst in mouse macrophages

**Fig. 1:**
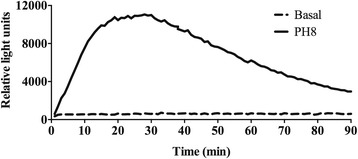
Production of reactive oxygen species by macrophages stimulated with *L. amazonensis*. Thioglycollate-elicited macrophages were harvested from the peritoneal cavity of C57BL/6 mice 3 days after stimulation. Macrophages were placed in plates with luminol and *L. amazonensis* metacyclic promastigotes were added (10 parasites per macrophage). Registration of light emission was performed for 90 min immediately after addition of *L. amazonensis*. Production of reactive oxygen species was measured as relative light units generated by luminol oxidation. Basal readings were obtained by adding luminol to macrophages from the same mouse without addition of *L. amazonensis.* Data are representative of three independent experiments, *n* = 4 for each replicate

ROS are very important for the elimination of a variety of intracellular pathogens. Hence, we first analysed if *L. amazonensis* metacyclic promastigotes triggered the respiratory burst in murine macrophages. We followed ROS production for 90 min using luminometry [40]. Macrophages produced ROS within two minutes of infection, maximum production was found between 15 and 30 min after addition of parasites (Fig. 1). Once it was determined that *L. amazonensis* metacyclic promastigotes could stimulate ROS production by macrophages, we proceeded to investigate if ROS would have an effect on the course of infection with this parasite.

### Gp91^phox−/−^ mice develop larger lesions after infection with L. amazonensis

To address the importance of ROS during *L. amazonensis* infection, we infected mice deficient in the gp91^phox^ subunit of NOX2 (gp91^phox−/−^). This subunit is responsible for transferring electrons from NADPH to oxygen, generating superoxide anion. Consequently, gp91^phox−/−^ mice cannot produce ROS through NOX2 [23].

**Fig. 2:**
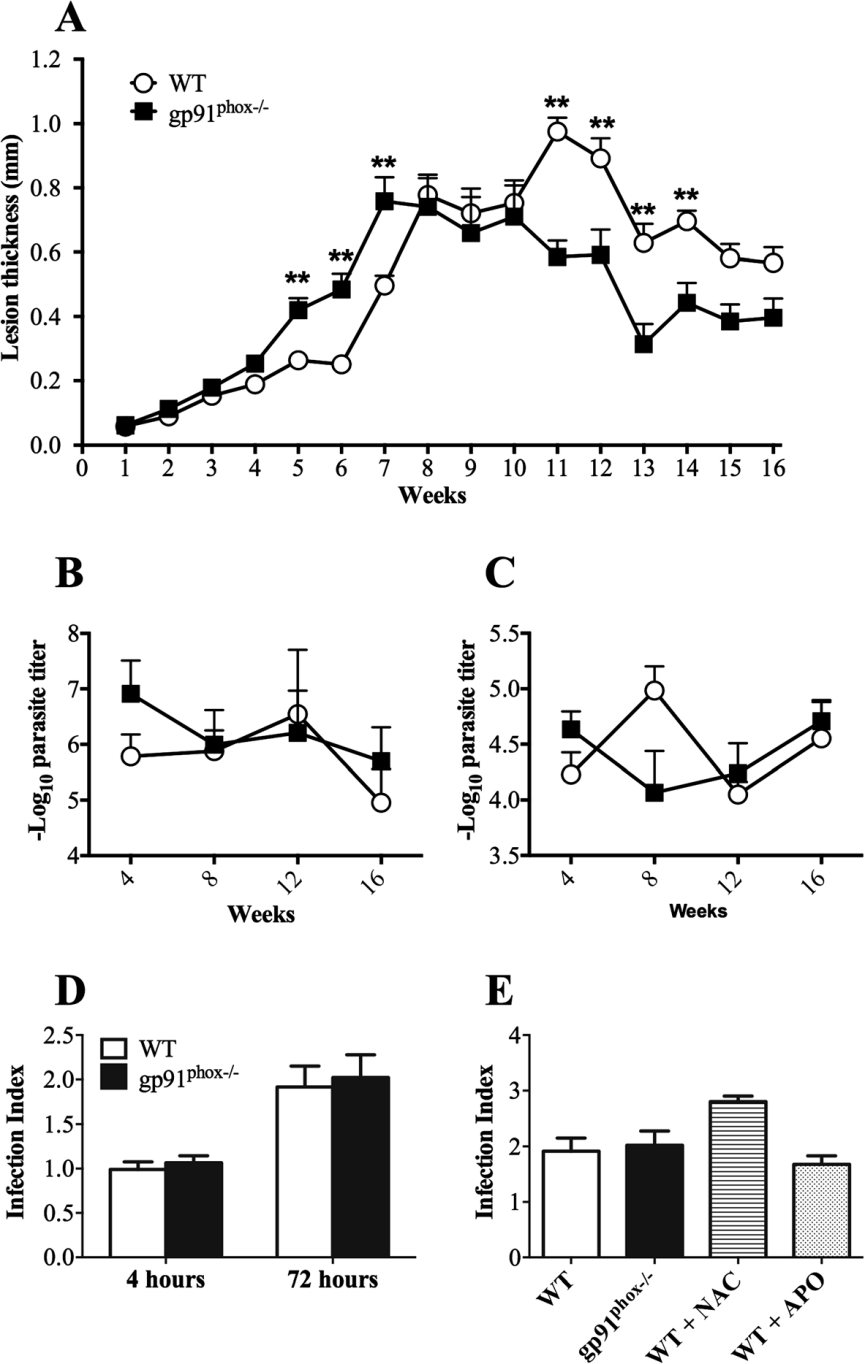
Lesion size and parasite loads in wild type (WT) and gp91^phox−/−^ mice infected with *L. amazonensis*. Mice were infected with 1 × 10^6^ metacyclic promastigote forms of *L. amazonensis* in the right hind footpad and followed until 16 weeks. **a** Footpad thickness, **P < 0.01 between WT and gp91^phox−/−^ mice. **b** Parasite loads in footpads of infected mice 4, 8, 12 and 16 weeks post-infection. **c** Parasite loads found in draining lymph nodes of infected mice 4, 8, 12 and 16 weeks post-infection. Data are shown as mean ± SD from one representative experiment of three, n = 5 for each experiment. **d** and **e** Inflammatory macrophages obtained from peritoneal cavity of C57BL/6 and gp91^phox−/−^ mice were infected with *L. amazonensis* metacyclic promastigotes. The cells were washed to remove extracellular parasites and either fixed or re-incubated with medium for 72 h. The slides were stained and counted to determine the infection index. A minimum of 200 macrophages were counted per group in triplicate. **d** Infection index for C57BL/6 and gp91^phox−/−^ macrophages four and 72 h post *in vitro* infection. **e** Infection index in macrophages from WT pre-incubated with N-acetyl-cysteine (NAC) or apocynin (APO) or from gp91^phox**−/−**^ mice 72 h post *in vitro* infection

We observed larger lesions in gp91^phox−/−^ mice from five to seven weeks post-infection with *L. amazonensis* (Fig. 2a), when compared to lesions in WT mice. However, 11 weeks post-infection, lesions in gp91^phox−/−^ started to decline and were significantly smaller than lesions in WT mice until week 14 of infection. Lesions in both groups declined and were similar at weeks 15 and 16 weeks post-infection.

Regardless of differences in lesion sizes observed between gp91^phox−/−^ and WT mice, we found no differences in parasite loads, neither in lesions nor in draining lymph nodes, at 4, 8, 12 and 16 weeks post-infection (Fig. 2b, c). In addition, we found no differences between *in vitro* infection of WT and gp91^phox−/−^ macrophages (Fig. 2d). Addition of apocynin, an inhibitor of NOX2, or N-acetyl-cysteine to WT macrophages confirmed that *L. amazonensis* is not susceptible to oxidative stress within macrophages (Fig. 2e).

### Cytokine production in footpads, draining lymph nodes and *in vitro*-infected macrophages

**Fig. 3:**
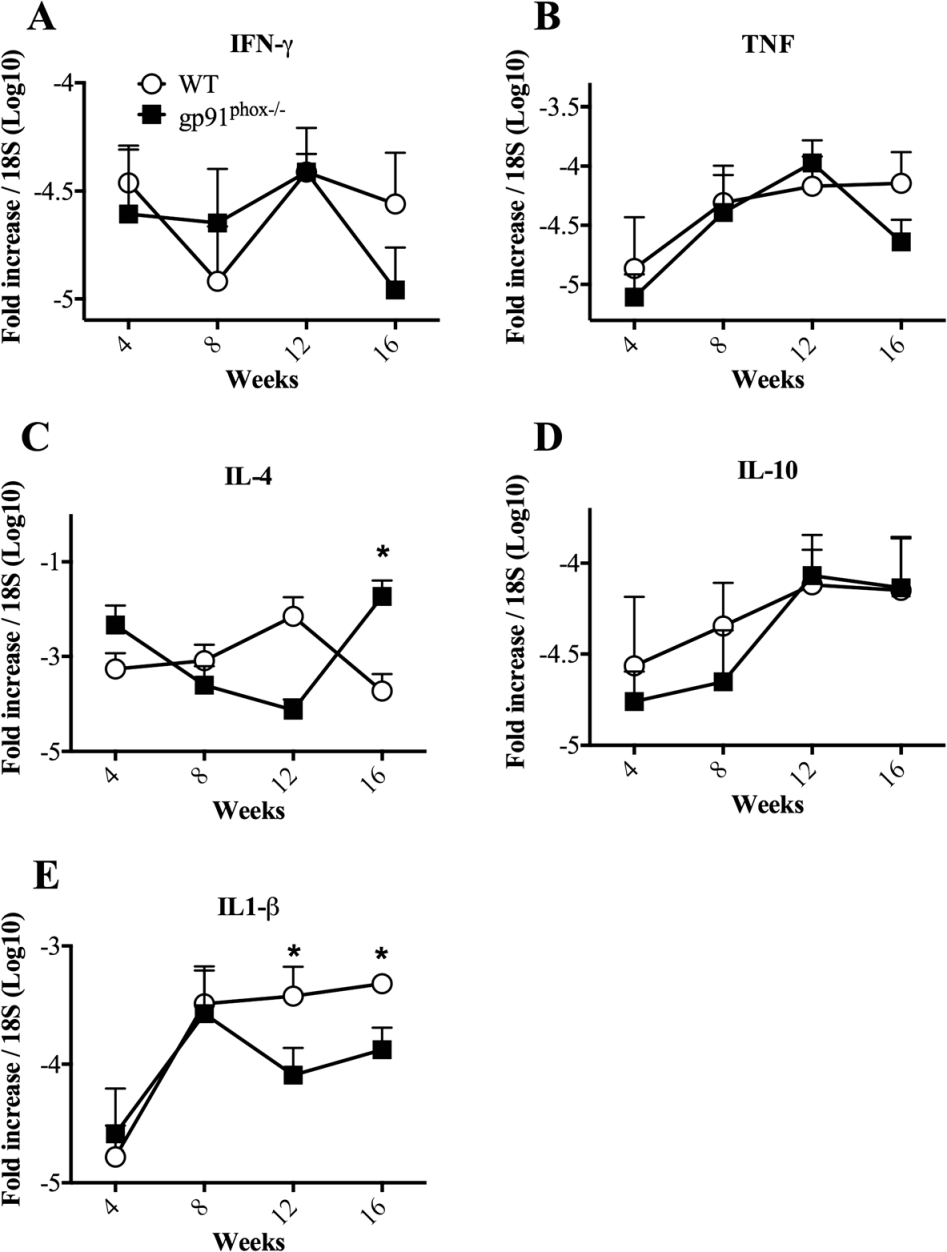
mRNA expression levels of cytokines from *L. amazonensis*-infected footpads measured by qPCR. Wild type (WT) and gp91^phox−/−^ mice were infected with 1 × 10^6^ metacyclic promastigote forms of *L. amazonensis* in the right hind footpad and followed for 16 weeks. **a**, **b**, **c**, **d** and **e** represent the mRNA expression of IFN-γ, TNF-α, IL-4, IL-10 and IL-1β, respectively, normalized by 18S mRNA expression in each time point. The results were expressed by mean ± SD, *n* = 5, *P < 0.05 between WT and gp91^phox−/−^ mice

Since IFN-γ and TNF-α are crucial to eliminate *Leishmania* in macrophages, we investigated the mRNA expression of these cytokines in gp91^phox−/−^ mice. No differences in mRNA levels for IFN-γ or TNF-α in footpads were detected in any of the times measured (Fig. 3a, b). However, we found higher expression of IL-1β mRNA in WT mice at 12 and 16 weeks of infection (Fig. 3e). Moreover, mRNA levels of IL-4 were higher in gp91^phox−/−^ at the last time point measured (Fig. 3c). No differences in IL-10 mRNA levels between groups were observed (Fig. 3d).

**Fig. 4:**
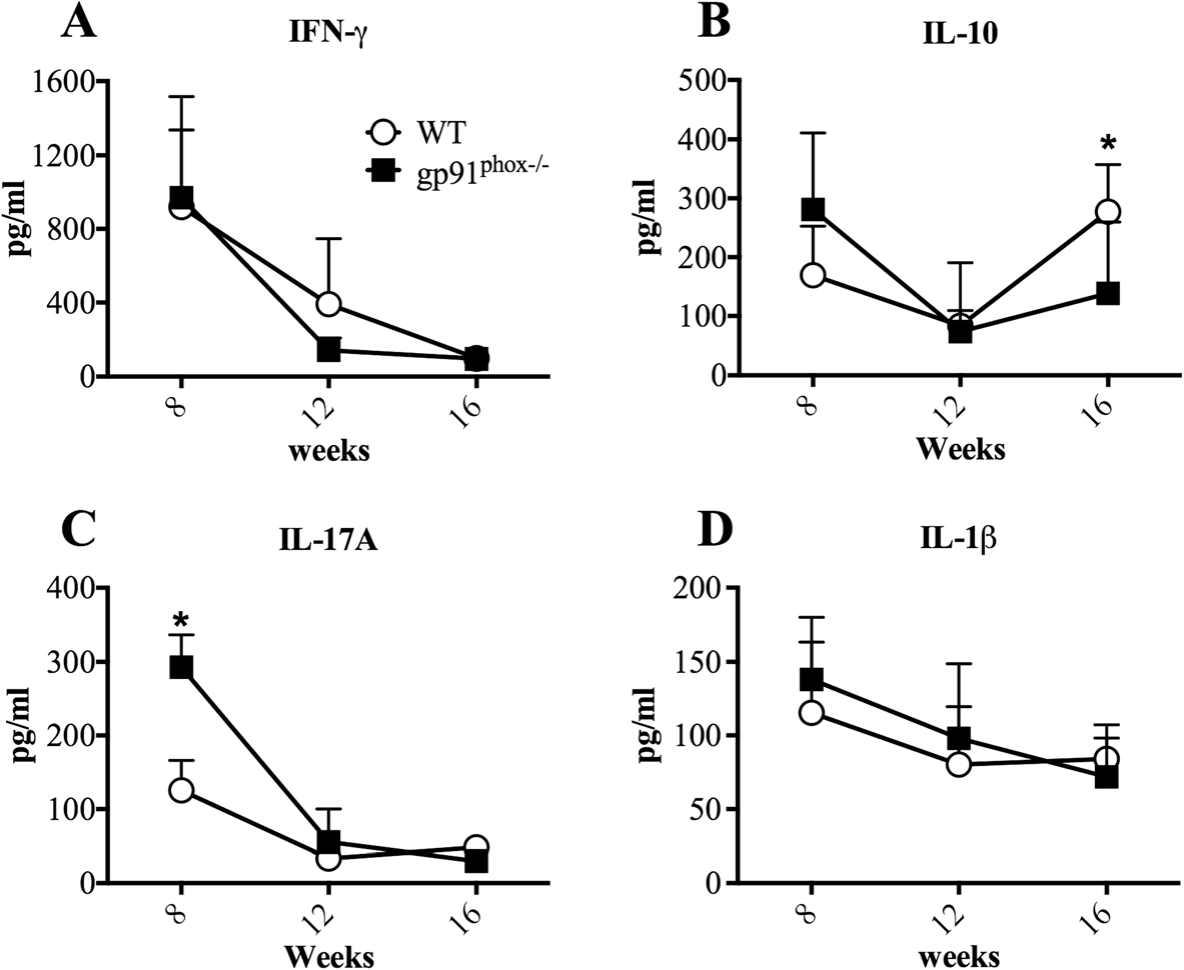
Cytokine production by re-stimulated draining lymph node cells from *L. amazonensis*-infected wild type (WT) and gp91^phox−/−^ mice. Draining lymph node cells were cultured and re-stimulated with 50 μg/ml of *L. amazonensis* antigen. After 72 h, the supernatants were harvested and used to quantify cytokines by ELISA. **a**, **b**, **c** and **d** represent the IFN-γ, IL-10, IL-17A and IL-1β secretion by draining lymph node cells, respectively. The results were expressed by mean ± SD, *n* = 5, *P < 0.05 between WT and gp91^phox−/−^ mice

We did not observe differences in IFN-γ production by draining lymph node (dLN) cells 8, 12 and 16 weeks post-infection (Fig. 4a). Larger production of IL-10 was found in gp91^phox−/−^ mice 16 weeks after infection (Fig. 4b).

Draining lymph node cells from gp91^phox−/−^ mice produced more IL-17A 8 weeks post-infection than cells from WT mice. IL-17 production by dLN cells dropped at 12 and 16 weeks post-infection, and no differences were found between the groups (Fig. 4c). No differences in IL-17 mRNA were found in lesions (data not shown). Interestingly, despite the differences found in IL-1β mRNA expression levels 12 and 16 weeks post-infection in WT infected footpads (Fig. 3e), we could not observe augmented levels of this cytokine in dLNs of WT in all periods measured when compared to gp91^phox−/−^ dLNs (Fig. 4d).

**Fig. 5:**
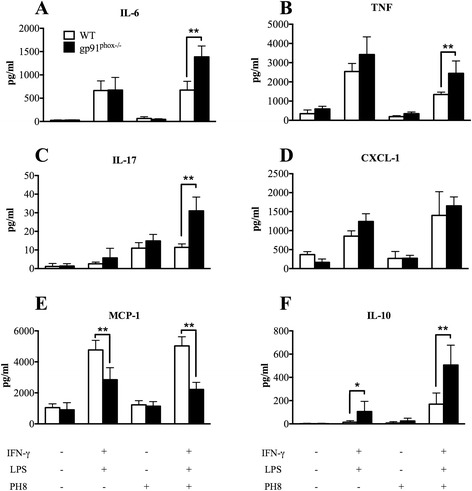
Cytokine production by macrophages infected with *L. amazonensis* *in vitro*. Thioglycollate-elicited macrophages were harvested from the peritoneal cavity of WT or gp91^phox−/−^ mice 3 days after stimulation. The cells were incubated  in 24-well plates for 16 h at 37 ^o^C, 5 % of CO_2_ and then washed for non-adherent cells removing. The adherent cells were stimulated or not with 50 U/ml of IFN-γ plus 100 ng/ml of LPS for 24 h at 37 ^o^C, 5 % of CO_2_. Macrophages were infected with *L. amazonensis* metacyclic promastigotes (10 parasites per macrophage) for 4 h and cultures were washed and re-stimulated or not with IFN-γ plus LPS at the same concentrations used before. After 48 h of infection supernatants were collected and used to measure cytokines by ELISA for IL-6 (**a**), TNF (**b**), IL-17 (**c**), CXCL-1 (**d**), MCP-1 (**e**) and IL-10 (**f**). Data are shown as mean ± SD of one representative experiment of four, *n* = 5 for each experiment

We also determined the production of cytokines by *in vitro*-infected macrophages (Fig. 5). We found that macrophages from gp91^phox−/−^ and WT mice produced similar levels of IL-6, TNF-α, IL-17A and CXCL-1, MCP-1 and IL-10, regardless of infection with *L. amazonensis*. However, upon stimulation with LPS (a stimulus for TNF-α production) and IFN-γ, infected macrophages from gp91^phox−/−^ mice produced higher levels of IL-6, TNF-α (albeit not consistently in all experiments), IL-17A and IL-10. CXCL-1 production was similar in both macrophages, while MCP-1 was higher in WT macrophages than in gp91^phox−/−^ cells.

### Deficiency in ROS production is not compensated by increase in iNOS expression

ROS and RNS may play complementary roles in killing intracellular parasites [32, 41]. However, in our experiments, gp91^phox−/−^ mice harboured parasites similarly to WT. This could be due to an irrelevant role of ROS in infection with *L. amazonensis* or to a compensatory over-production of nitric oxide. We investigated the mRNA expression of inducible nitric oxide synthase (iNOS) in lesions and the production of nitric oxide (NO) by peritoneal macrophages in gp91^phox−/−^ mice.

**Fig. 6:**
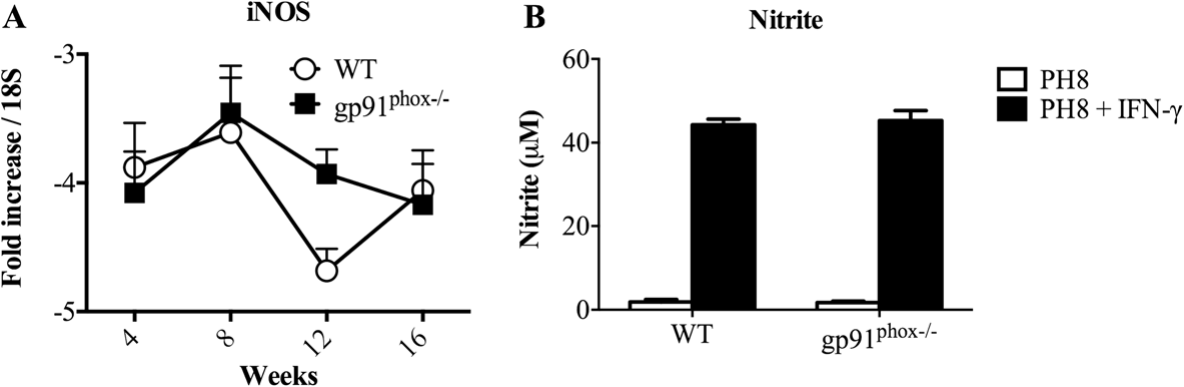
iNOS mRNA expression in footpads and nitrite production by infected thioglycollate-elicited macrophages. **a** Wild type (WT) and gp91^phox−/−^ mice were infected with 1 × 10^6^ metacyclic promastigote forms of *L. amazonensis* in the right hind footpad and followed for 16 weeks. iNOS mRNA levels were normalized by 18S mRNA expression 4, 8, 12 and 16 weeks post-infection. **b** Thioglycollate-elicited macrophages were harvested from the peritoneal cavity of WT or gp91^phox−/−^ mice 3 days after stimulation. Macrophages were infected with *L. amazonensis* metacyclic promastigotes (10 parasites per macrophage) for 4 h and cultures were washed. After 48 h of infection supernatants were collected and used to measure nitrite levels by Griess reaction. Data are shown as mean ± SD of one representative experiment of four, *n* = 5 for each experiment

Messenger-RNA levels of nitric oxide in lesions were similar between groups at all times measured. Both groups displayed higher expression of iNOS at 8 weeks post-infection, and lower levels of expression 4, 12 and 16 weeks post-infection (Fig. 6a). Thus, ROS deficiency did not increase the transcription of iNOS genes. In addition, *in vitro*-infected IFN-γ-activated macrophages from gp91^phox−/−^ and WT mice produced similar levels of NO (Fig. 6b).

### ROS influence the migration of neutrophils after infection with *L. amazonensis*

**Fig. 7:**
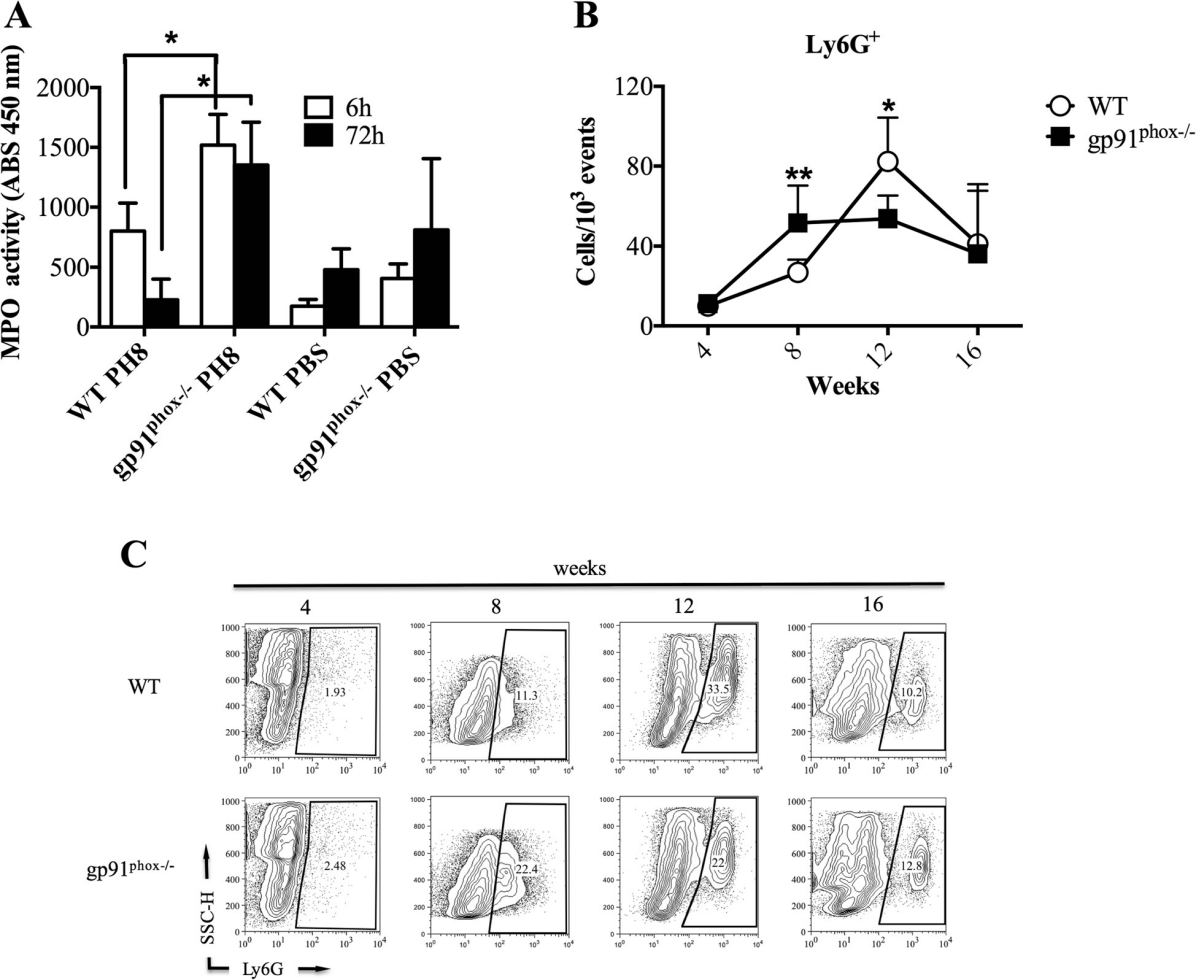
Myeloperoxidase (MPO) activity and Ly6G^+^ cells at the infection site in wild type (WT) and gp91^phox−/−^ mice. Mice were infected with 1 × 10^6^ metacyclic promastigote forms of *L. amazonensis* (PH8) in the right hind footpad, or injected with PBS. **a** After 6 h or 72 h of infection, footpads were removed and used to assay MPO activity to estimate neutrophil numbers in the acute phase of infection. **b** Flow cytometry of Ly6G^+^ cells in footpads 4, 8, 12 and 16 weeks post-infection, performed as described in the Methods section. Data are shown as mean ± SD from one representative experiment of two, *n* = 5 for each experiment, *P < 0.05, **P < 0.01 between WT and gp91^phox−/−^ mice. **c** Representative dot plots of Ly6G^+^ cells recovered from infected footpads after 4, 8, 12 and 16 weeks of infection. The dot plots represent percentage of Ly6G^+^ cells gated on side scatter versus forward scatter cell properties

Neutrophils are important to eliminate invading microorganisms, and one of the mechanisms of this elimination is by ROS production. Hence, we investigated neutrophil migration to the site of infection in gp91^phox−/−^ and WT mice. First, we infected mice with 1 × 10^6^ *L. amazonensis* metacyclic promastigotes in the footpads and analysed the accumulation of neutrophils indirectly by the detection of myeloperoxidase (MPO) activity, 6 h and 72 h after infection. Due to the higher expression of MPO in granulocytes than in other leukocytes, it is possible to estimate the quantity of neutrophils present in the tissue. Neutrophils accumulated in footpads in WT mice, but were not found in higher density 72 h post-infection (Fig. 7a). In gp91^phox−/−^ mice we found more MPO activity 6 h after infection. More interestingly, MPO activity persisted 72 h after infection (Fig. 7a). We also followed the accumulation of granulocytes throughout the course of infection by flow cytometry, using the Ly6G marker. At eight weeks post-infection there were more neutrophils in footpads from gp91^phox−/−^ mice (Fig. 7b, c). On the other hand, there were more neutrophils at 12 weeks post-infection in WT mice.

## Discussion

In this work, we analysed the importance of ROS production by the host on the outcome of infection caused by *L. amazonensis*. C57BL/6 mice are unable to spontaneously heal lesions after subcutaneous infection with *L. amazonensis*, but develop chronic lesions that do not progress indefinitely [6, 8]. We observed increase in paw swelling of infected WT mice until week 7 post-infection, followed by a slight decrease and lesion stabilization after this time point. Indeed, our results are supported by other works showing similar lesion kinetics during *L. amazonensis* infection in C57BL6 mice [42–44]. A role for IFN-γ and iNOS in this partial resistance to the parasite is clear, especially at later times of infection, since *L. amazonensis-*infected IFN-γ^−/−^ and iNOS^−/−^ mice develop larger non-healing lesions than wild type mice [44, 45]. ROS have been shown to play a role in the containment of metastasis to spleen and lymph nodes in experimental *L. major* infection [30]. Macrophages [29, 30] and neutrophils [30] respond to infection with *L. major* with ROS production, as shown *in vitro*. However, killing of *L. major* by IFN-*γ*-activated macrophages is dependent on NO production, but not on the production of superoxide or peroxynitrite [29], hampering the role of ROS in resistance to this parasite. *L. guyanensis* amastigotes, on the other hand, die inside BALB/c macrophages through an apoptosis-like process mediated by parasite-induced ROS [31]. However, little is known about the role of ROS in resistance to *L. amazonensis*. It has been reported that *L. amazonensis* triggers less ROS production by macrophages from CBA mice *in vitro* 30 min after infection [33]. However, production of ROS by activated macrophages from C57BL/6 mice infected with *L. amazonensis* 30 min and 5 days post-infection has been reported [46]. Production of ROS was dependent on gp91^phox^ and ROS mediated parasite killing *in vitro*. In addition, *in vivo* ROS production by C57BL/6 mice during *L. amazonensis* infection has been reported [47, 48]. The production of ROS was determined indirectly by detection of nitrotyrosine, which provides evidence of peroxynitrite production. Peroxynitrite is produced by the reaction of ROS (produced by NADPH oxidase) and NO (produced by iNOS), these enzymes co-localize in the phagolysosome, speaking for their role in the production of peroxynitrite and damage to the parasite [47].

In our experiments, using metacyclic promastigote forms of PH8 *L. amazonensis* strain and thioglycollate-elicited macrophages from C57BL/6 mice, *L. amazonensis* induces ROS production by peritoneal macrophages *in vitro* (Fig. 1). Parasites of the genus *Leishmania* possess several mechanisms to escape ROS in phagolysosomes. *L. pifanoi* is capable of blocking the maturation of the gp91^phox^ subunit of RAW 264.7 cells, avoiding the NADPH oxidase complex formation [34]. *L. donovani* LPG blocks the translocation of p47^phox^ and p67^phox^ to the phagosome, inhibiting NADPH oxidase activation [49]. *L. amazonensis* promastigotes developed strategies to resist to ROS; however, the mechanisms of this resistance have not been determined yet. It is possible that *L. amazonensis* does not inhibit NOX2, since *L. amazonensis-*infected CBA macrophages respond to infection with *L. major* similarly to uninfected macrophages [33]. On the other hand, the host produces ROS in response to a variety of stimuli, such as activated complement [50], binding to IgG Fc receptor (FcγR) [51] and Toll-like receptor 2 [17], during *Leishmania* spp. infection. Considering the sophisticated mechanisms used by the parasites to escape damage by ROS and the variety of mechanisms by which the host achieves ROS production, allied with the susceptibility of some species of *Leishmania* to ROS [52], it was reasonable to infer that ROS may play a role in resistance to *L. amazonensis.* Lesion development in gp91^phox−/−^ and WT mice after infection with *L. amazonensis* was quite different. In the first seven weeks of infection, gp91^phox−/−^ mice presented larger lesions, but the same number of parasites as WT mice. Coherently, macrophages from WT and gp91^phox−/−^ mice harboured similar numbers of parasites and produced similar levels of NO when activated with IFN-γ, and similar levels of message for iNOS were found in footpads from both infected mouse strains. In addition, cytokine levels at the site of infection, as well as IFN-γ, IL-1β and IL-10 production by antigen-stimulated lymph node cells were similar among groups. However, IL-17 production by draining lymph node cells was higher in gp91^phox−/−^ mice than in WT at 8 weeks of infection. Interestingly, MPO activity was higher in gp91^phox−/−^ footpads than in WT at 6 and 72 h post-infection. In addition, more Ly6G^+^ cells were found at the site of infection 8 weeks post-infection in gp91^phox−/−^ mice, subsequently to when gp91^phox−/−^ mice displayed larger lesions than WT mice. On the other hand, at 12 weeks of infection, when WT mice had larger lesions, they also had more neutrophils at the site of infection. These data suggest that ROS could have a relevant role in modulating the inflammatory infiltrate, but irrelevant action in parasite killing *in vivo*. The reason for the larger lesions up to 7 weeks (maybe coinciding with larger numbers of neutrophils) and smaller lesions at later time points (also coinciding with smaller numbers of neutrophils) is not yet known. Ablation of neutrophils early in infection with *L. amazonensis,* in our experiments, caused no effect in the outcome of infection in C57BL6 mice [53]. However, our results here suggest that the persistence of this otherwise short-lived cell may exacerbate pathology. Larger neutrophil infiltrates are found in NOX2-deficient mice [23], and that would explain, possibly, the larger lesions at the earlier time points of infection. As stated above, at the later time points a larger neutrophil infiltrate was found in WT mice. The reason for the variation in these numbers in the two mice is unknown at this point. We could, however, speculate that the neutrophil infiltrate is more precocious in gp91^phox−/−^ mice and more precociously resolved than in WT mice. Eventually, lesions were the same in both mouse strains.

Humans deficient on functional NADPH oxidase develop chronic granulomatous disease (CGD), which is characterized by recurrent infections and limitations in elimination of intracellular microorganisms; gp91^phox−/−^ mice kept in less sanitary conditions may also develop CGD [23, 25]. Moreover, gp91^phox−/−^ mice show high numbers of neutrophils during peritonitis caused by chemical agents [23] as well as increased inflammatory cytokine and chemokine production [26]. There are solid evidences implicating ROS in the induction of neutrophil apoptosis [54–60]. Consequently, it is possible to infer that a decrease in neutrophil apoptosis culminates with the hyper-inflammation seen in CGD [56, 61–64]. Accordingly, at early time points (6 and 72 h, Fig. 7a) we observed higher numbers of neutrophils at the infection site in gp91^phox−/−^ mice. This fact could be related with the impaired capacity of neutrophils from gp91^phox−/−^ mice to start the apoptotic programme *via* ROS. Moreover, other mechanisms such as persistent cellular activation [65], attenuation of ROS dependent Ca^2+^ signaling [66, 67] and oxidation of transcriptional factors and phosphatases via ROS [68] could contribute to the larger lesions in gp91^phox−/−^ mice. Despite extensive data in the literature reporting increased expression of inflammatory cytokines in gp91^phox−/−^ mice [66, 67, 69, 70], we could not detect alterations in cytokine mRNA levels (Fig. 3a, b) in lesions. Nevertheless, macrophages infected *in vitro* with *L. amazonensis* and activated with IFN-γ produced higher levels of IL-6, TNF-α, IL-17A and IL-10 (perhaps in response to high levels of TNF-α [71]) and lower levels of MCP-1 when compared to cytokine production by WT macrophages. In addition, a larger production of IL-17A by lymph node cells was found at 8 weeks of infection, which correlated with the larger neutrophil infiltrate. Th17 response is related to increased neutrophil migration in many experimental models [72–75]. The higher production of IL-17A observed herein could contribute to the migration of neutrophils to lesions and promote a larger inflammatory infiltrate in gp91^phox−/−^ mice. Surprisingly, after 11 weeks of infection, lesions in gp91^phox−/−^ mice started to decrease and became smaller that lesions in the WT group. Again, no differences in parasite numbers were found between groups. However, the mRNA levels IL-1β were higher in WT mice at later time points, indicating an inflammatory status. Indeed, IL-1β expression could be dependent of ROS release [76], which could explain the decrease of footpad swelling and the low concentration mRNA levels of IL-1β observed in gp91^phox−/−^ mice at this time point. Gp91^phox−/−^ footpads expressed higher mRNA levels for IL-4, which could indicate an anti-inflammatory status at later times of infection. No differences in IL-10 levels were found throughout infection.

## Conclusion

To the best of our knowledge, this is the first report addressing the role of NOX2 in experimental murine infection with *L. amazonensis*. Our results indicate that ROS might regulate the inflammation caused in *L. amazonensis* infection, but does not affect parasite control. The mechanisms used by *L. amazonensis* to avoid killing by ROS still need to be addressed.
